# Comprehensive Analyses and Immunophenotyping of LIM Domain Family Genes in Patients with Non-Small-Cell Lung Cancer

**DOI:** 10.3390/ijms24054524

**Published:** 2023-02-24

**Authors:** Sini Li, Lihui Liu, Yan Qu, Li Yuan, Xue Zhang, Zixiao Ma, Hua Bai, Jie Wang

**Affiliations:** 1 National Cancer Center/National Clinical Research Center for Cancer/Cancer Hospital, Chinese Academy of Medical Sciences and Peking Union Medical College, Beijing 100021, China; 2 CAMS Key Laboratory of Translational Research on Lung Cancer, State Key Laboratory of Molecular Oncology, Department of Medical Oncology, National Cancer Center/National Clinical Research Center for Cancer/Cancer Hospital, Chinese Academy of Medical Sciences and Peking Union Medical College, Beijing 100021, China

**Keywords:** LIM domain family, LIMS1, molecular subtypes, non-small-cell lung cancer, tumor microenvironment

## Abstract

The LIM domain family genes play a crucial role in various tumors, including non-small-cell lung cancer (NSCLC). Immunotherapy is one of the most significant treatments for NSCLC, and its effectiveness largely depends on the tumor microenvironment (TME). Currently, the potential roles of LIM domain family genes in the TME of NSCLC remain elusive. We comprehensively evaluated the expression and mutation patterns of 47 LIM domain family genes in 1089 NSCLC samples. Using unsupervised clustering analysis, we classified patients with NSCLC into two distinct gene clusters, i.e., the LIM-high group and the LIM-low group. We further investigated the prognosis, TME cell infiltration characteristics, and immunotherapy in the two groups. The LIM-high and LIM-low groups had different biological processes and prognoses. Moreover, there were significant differences in TME characteristics between the LIM-high and LIM-low groups. Specifically, enhanced survival, immune cell activation, and high tumor purity were demonstrated in patients of the LIM-low group, implying an immune-inflamed phenotype. Moreover, the LIM-low group had higher immune cell proportion scores than the LIM-high group and was more responsive to immunotherapy than the LIM-low group. Additionally, we screened out LIM and senescent cell antigen-like domain 1 (LIMS1) as a hub gene of the LIM domain family via five different algorithms of plug-in cytoHubba and the weighted gene co-expression network analysis. Subsequently, proliferation, migration, and invasion assays demonstrated that LIMS1 acts as a pro-tumor gene that promotes the invasion and progression of NSCLC cell lines. This is the first study to reveal a novel LIM domain family gene-related molecular pattern associated with the TME phenotype, which would increase our understanding of the heterogeneity and plasticity of the TME in NSCLC. LIMS1 may serve as a potential therapeutic target for NSCLC.

## 1. Introduction

Lung cancer is one of the most common cancers worldwide, which seriously endangers public health [1]. Based on the Global Burden of Disease Study in 2020, lung cancer has the second highest incidence and the highest mortality [2], of which non-small-cell lung cancer (NSCLC) accounts for approximately 80−85% [3,4]. Owing to the high invasiveness of NSCLC and the lack of significant clinical symptoms in early-stage patients, most patients with NSCLC are already in the advanced stage at the time of diagnosis, with a relatively poor prognosis and high mortality [5]. Although targeted therapy and immunotherapy have primarily improved the survival of patients with NSCLC, some patients do not respond to these treatments [6,7] owing to the molecular heterogeneity of tumors [8]. Therefore, an in-depth understanding of tumor characteristics and the identification of effective prognostic indicators are needed to develop individualized diagnosis and treatment.

The LIM domain family is a specialized tandem zinc-finger structure recognized as a modular protein-binding interface [9]. The LIM domain family has been identified in both the cytoplasm and the nucleus and consists of many members, including members of the LIM homeobox (LHX), C-reactive protein (CRP), four-and-a-half LIM protein (FHL), Paxillin, LIM Domain Kinase (LIMK), LIM-only (LMO), Enigma, microtubule-associated oxygenase, calponin and LIM domain (MICAL), LIM and SH3 protein (LASP), actinin-associated LIM protein (ALP), particularly interesting new Cys-His protein (PINCH), Testin, and Zyxin families [10]. Increasing evidence reveals that the LIM domain family has diverse functions in regulating cytoskeleton organization, tissue-specific gene expression, neuronal pathfinding, cell fate determination, cell adhesion, cell motility, and signal transduction [11,12]. Moreover, it is emerging as a critical molecule in a wide variety of human cancers. The LMO proteins have important roles in cancer initiation and progression [13], and PINCH has been reported to promote tumor progression and metastasis [14,15], suggesting that the LIM domain family may be a potential therapeutic target for a range of different cancers.

It has been recently reported that the tumor microenvironment (TME) is closely related to biological processes during tumorigenesis, including tumor initiation, progression, metastasis, and immune escape [16,17]. Immunophenotyping of tumors is crucial in formulating effective treatment strategies, especially immunotherapy, and is significant in the prognostic assessment of patients with tumors [18]. Increasing evidence has revealed the correlation between TME infiltrates and the LIM domain family genes. The LIM and senescent cell antigen-like domain 1 (LIMS1), a member of PINCH, was positively associated with advanced TNM stage and poor prognosis of patients with pancreatic cancer and promoted cancer cell survival in the oxygen-glucose-deprived TME [19]. The high expression of PDZ and LIM domain 2 (PDLIM2), a member of ALP, was also correlated with infiltrating immune cells and predicted poor prognoses in patients with prostate cancer [20]. However, the gene signature associated with the LIM domain family genes and its potential roles in immune infiltration remains elusive, especially in NSCLC. Therefore, immunophenotyping of LIM-mediated TME may aid in the treatment and prognosis of patients with NSCLC.

## 2. Results

### 2.1. The Genetic Landscape of the LIM Domain Family Genes in NSCLC

**Figure 1:**
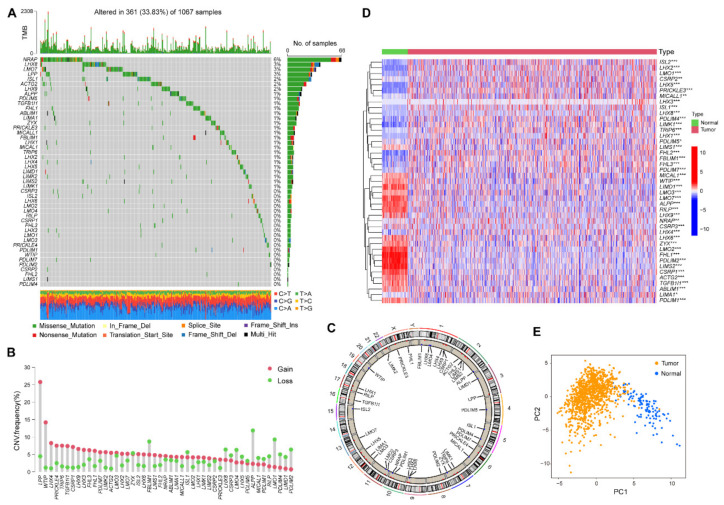
The genetic landscape of the LIM domain family genes in non-small-cell lung cancer (NSCLC). (**A**) Mutation frequencies of 47 LIM domain family genes in 1067 patients with NSCLC in The Cancer Genome Atlas. Each column indicates one patient. The top bar indicates tumor mutation burden. The numbers on the right represent the mutation frequency in each gene. The right bar indicates the proportion of each variant type. The stacked bar indicates the fraction of conversions in each sample. (**B**) The CNV alternations of 47 LIM domain family genes in NSCLC. The red dot indicates amplifications, and the green dot represents deletions. The height of the column suggests the alteration frequency. (**C**) The location of CNV alterations of LIM domain family genes. (**D**) The heatmap of differentially expressed LIM domain family genes between NSCLC and normal tissues. Low expression, blue; high expression, red. * *p* < 0.05; ** *p* < 0.01; *** *p* < 0.001. (**E**) Principal component analysis for 43 differentially expressed LIM domain family genes to distinguish samples in NSCLC and normal tissues.

First, a total of 47 genes in the LIM domain family were identified, including members of the *LHX*, *CRP*, *FHL*, *Paxillin*, *LIMK*, *LMO*, *Enigma*, *MICAL*, *LASP*, *ALP*, *PINCH*, *Testin*, and Zyxin families (Table 1). Second, we analyzed the somatic mutation frequency and copy number variations of 47 LIM domain family genes in NSCLC. Among 1067 samples with somatic mutation data, 361 exhibited LIM domain family gene mutations, with a frequency of 33.83%. Nebulin-related anchoring protein exhibited the highest mutation frequency, followed by *LHX8*. In contrast, some LIM domain family genes did not exhibit any mutation in NSCLC samples, including cysteine and lysine-rich protein 3 (*CSRP3*), ISL LIM homeobox 2 (*ISL2*), and *LHX6* (Figure 1A). The investigation of copy number variation (CNV) alteration frequency revealed that the LIM family genes exhibited prevalent CNV alterations, most of which were copy number amplifications. However, filamin binding LIM protein 1 (*FBLIM1*), alkaline phosphatase placental type (*ALPP*), *LMO1*, *PDLIM2*, *PDLIM4*, and LIM domain-containing protein 1 had a widespread frequency of CNV deletion (Figure 1B). The location of CNV alterations of the LIM domain family genes on chromosomes is presented in Figure 1C. To determine the relationship between the expression of LIM family genes and lung cancer, we explored mRNA levels of these genes in NSCLC and normal tissues. According to the results, 43 of the 47 LIM family genes were differentially expressed in NSCLC compared to normal tissues (Figure 1D). Moreover, according to the expression pattern of these genes, NSCLC samples were markedly distinct from normal samples (Figure 1E). These results revealed that the expression of the LIM domain family genes in NSCLC and normal tissues is different, indicating that the LIM domain family genes may play a potential role in the tumorigenesis of NSCLC.

### 2.2. LIM Signature Identified NSCLC with Distinct Prognoses and Biological Functions

**Figure 2:**
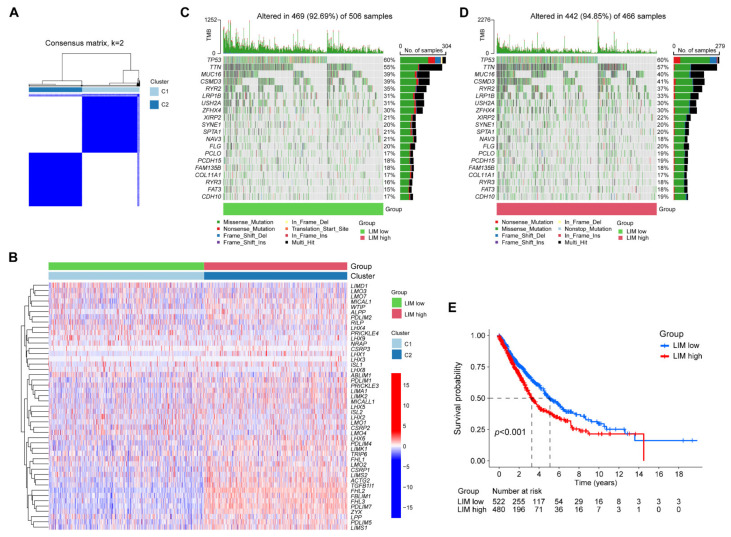
Identification of LIM domain family gene-related clusters in non-small-cell lung cancer (NSCLC). (**A**) According to the consensus cluster analysis, NSCLC samples were classified into two distinct gene clusters, with the optimal k = 2. (**B**) Unsupervised clustering of 47 LIM domain family genes in patients with NSCLC. The waterfall plots of tumor somatic mutation of the LIM-low group (**C**) and the LIM-high group (**D**). (**E**) Kaplan–Meier overall survival curves for the two groups in patients with NSCLC.

We explored the clinical significance of the LIM domain family genes in patients with NSCLC. Consensus clustering analysis was performed to classify patients with NSCLC into subgroups based on the expression of 47 LIM domain family genes. A total of 1002 patients with NSCLC were grouped into two clusters, including 522 and 480 cases in cluster 1 and cluster 2, respectively (Figure 2A, Table S1). Interestingly, we observed that most LIM domain family genes were more highly expressed in cluster 2 (LIM-high group) than in cluster 1 (LIM-low group, Figure 2B). Additionally, we explored the distribution of somatic mutations between the LIM-low group and the LIM-high group of patients with NSCLC. We did not observe a significant difference in mutation rates of LIM genes between the two groups (Figure 2C,D). However, survival analysis revealed that patients in the LIM-low group were associated with a significant survival benefit compared to those in the LIM-high group (Figure 2E).

To demonstrate the underlying biological pathways in the LIM-high group and the LIM-low group, we identified 863 differentially expressed genes (DEGs) between the two groups (Table S2). The Gene Ontology (GO) and Kyoto Encyclopedia of Genes and Genomes (KEGG) enrichment analyses were further performed based on DEGs to identify potential mechanisms (Tables S3 and S4). The GO analysis regarding biological processes demonstrated that DEGs were significantly enriched in extracellular matrix (ECM) organization and extracellular structure organization. In terms of cellular components and molecular function, DEGs were significantly enriched in the collagen-containing ECM and ECM structural constituents, respectively (Figure S1A). Additionally, the KEGG analysis revealed that DEGs were significantly enriched in protein digestion and absorption and ECM–receptor interaction, indicating the correlation with tumorigenesis and progression (Figure S1B). A Gene Set Enrichment Analysis (GSEA) was also performed to identify the functional enrichment of the LIM-high group and the LIM-low group of patients with NSCLC. As presented in Figure S1C–F and Table S5, the LIM-high group was prominently enriched in ECM–receptor interaction and cell–matrix adhesion (Figure S1C,D). However, the LIM-low group was markedly enriched in metabolic-related activities, including drug metabolism cytochrome P450 and olefinic compound metabolic process (Figure S1E,F).

### 2.3. Characterization of Tumor Immunophenotype with LIM Signature

**Figure 3:**
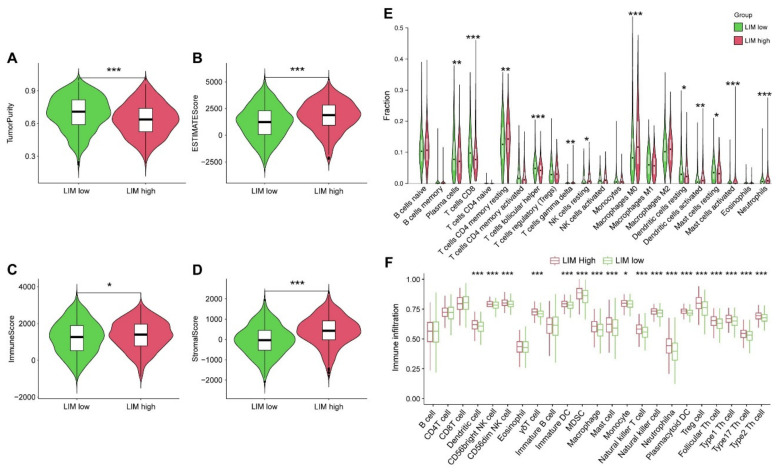
Tumor microenvironment (TME) characteristics of the LIM-high and the LIM-low groups. (**A**–**D**) The tumor purity, estimate score, immune score, and stromal score in the LIM-high and the LIM-low groups. The levels of TME infiltrating immune cells in the LIM-low and the LIM-high groups using cibersort (**E**) and ssGSVA algorithm (**F**). * *p* < 0.05; ** *p* < 0.01; *** *p* < 0.001.

To characterize the TME features of the LIM-high group and the LIM-low group of patients, we first explored the abundance of infiltrating immune cells via an estimate algorithm. Interestingly, the LIM-low group had higher tumor purity (Figure 3A) and lower ESTIMATEScore, ImmuneScore, and StromalScore (Figure 3B–D) than the LIM-high group. These results indicated heterogeneity in TME between the patients of the two groups. To further explore the TME features in detail, we compared the component differences of 22 types of immunocytes in two groups using CIBERSORT and ssGSEA analyses (Figure 3E,F, Tables S6 and S7). Consistently, we observed significant differences in the infiltration of immunocytes in TME between the two groups. Specifically, the LIM-low group had a higher percentage of CD8^+^ T cells, follicular helper T cells, resting dendritic cells, and resting mast cells than the LIM-high group. However, the proportion of CD4^+^ resting memory T cells, resting NK cells, M0 macrophages, activated dendritic cells, activated mast cells, and neutrophils were lower in the LIM-low group than those in the LIM-high group (Figure 3E). Additionally, ssGSEA analysis also revealed that myeloid-derived suppressor cells (MDSCs), macrophages, regulatory T cells, and mast cells were significantly more abundant in the LIM-high group than in the LIM-low group (Figure 3F). Altogether, these results demonstrated that the TME features and immune status of the two molecular subtypes significantly differed.

**Figure 4:**
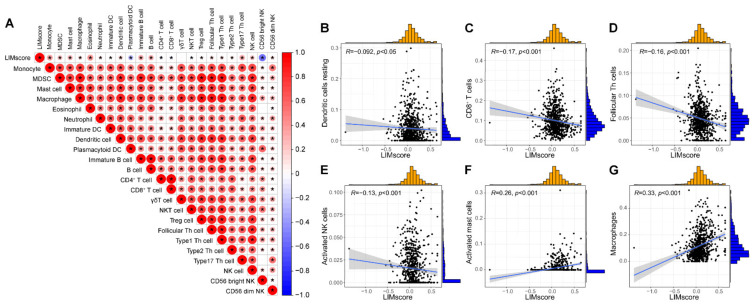
The LIMscore correlated with immune cell infiltration. (**A**) Correlations between LIMscore and immune cells. The LIMscore was negatively associated with the abundance of resting dendritic cells (**B**), CD8^+^ T cells (**C**), follicular helper T cells (**D**), and activated NK cells (**E**), albeit positively associated with activated mast cells (**F**) and macrophages (**G**). * *p* < 0.05

To further explore the correlation between the LIM signature and TME, we constructed a LIMscore algorithm using principal component analysis (PCA) based on the expression levels of 47 LIM domain family genes. By quantifying the LIMscore in each patient with NSCLC, we investigated the relationship between the LIMscore and infiltrating immune cells (Figure 4A). Interestingly, we observed that the LIMscore was negatively associated with the abundance of resting dendritic cells (Figure 4B), CD8^+^ T cells (Figure 4C), follicular helper T cells (Figure 4D), and activated NK cells (Figure 4E). A remarkable positive association was achieved between the LIMscore and the abundance of activated mast cells (Figure 4F) and macrophages (Figure 4G). These results demonstrated that a higher LIM signature may correlate with an immunosuppressive TME in patients with NSCLC.

### 2.4. Relationship between LIM Signature and Tumor Somatic Mutation and Immunotherapy

**Figure 5:**
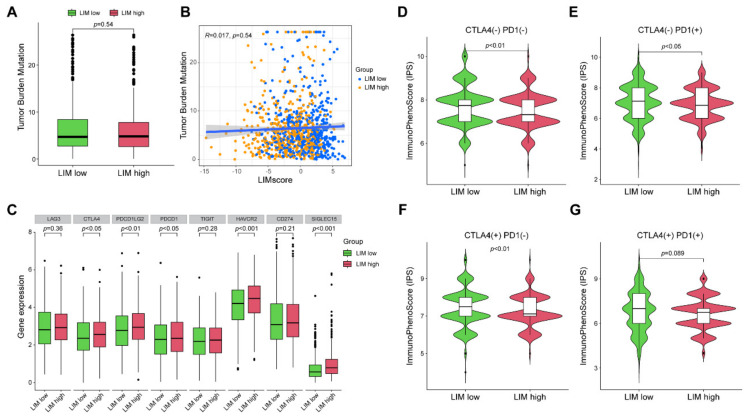
Relationship of LIM signature with tumor somatic mutation and immunotherapy. (**A**) The tumor mutation burden (TMB) of the LIM-low and the LIM-high groups. (**B**) Correlations between LIMscore and TMB. (**C**) The expression levels of immune checkpoints in the LIM-low and the LIM-high groups. (**D**–**G**) The immunotherapy response between the LIM-low and the LIM-high groups.

Considering that immune checkpoint inhibitors enhance the treatment pattern and provide significant benefits to patients with NSCLC, we investigated the potential relationship between the LIM signature and immunotherapy. First, we observed no significant difference in the tumor mutation burden (TMB) between the LIM-low and LIM-high groups (Figure 5A). Consistently, correlation analysis confirmed that the LIMscore was not associated with the TMB level (Figure 5B). Second, we explored the expressions of several widely used immune checkpoints in the LIM-high and LIM-high groups. As indicated in Figure 5C, compared with those in the LIM-low group, the expressions of cytotoxic T-lymphocyte associated protein 4 (CTLA4), programmed cell death (PCD) 1 ligand 2, PCD protein 1 (PDCD1), Hepatitis-A virus cellular receptor 2, and sialic acid binding Ig like lectin 15 were significantly increased in the LIM-high group. Additionally, we calculated the immune cell proportion score (IPS) for each sample and distinguished beneficiaries between the two groups. The IPSs of CTLA4_neg_PD1_neg, CTLA4_neg_PD1_pos, CTLA4_pos_PD1_neg, and CTLA4_pos_PD1_pos (Figure 5D–G) were significantly higher in the LIM-low group than those in the LIM-high group, suggesting more beneficiaries in the LIM-low group. These results indicated that the LIM-low group had more immunogenic phenotypes, and the LIM signature was intensively associated with immunotherapy.

### 2.5. Identifying LIMS1 as a Hub Gene in the LIM Domain Family

**Figure 6:**
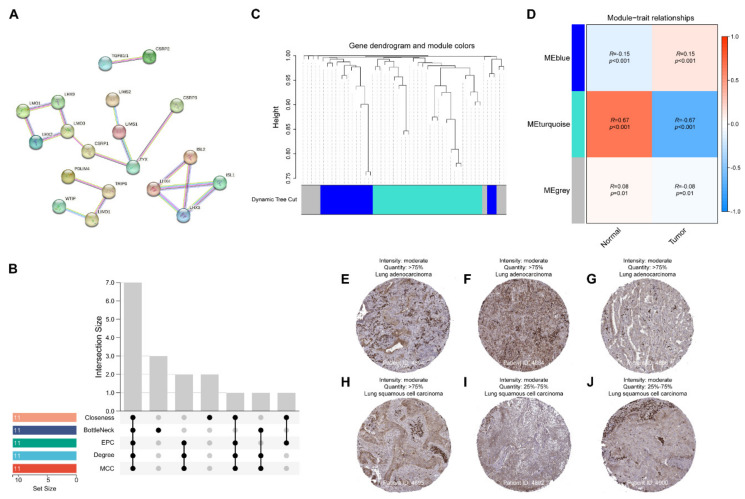
Hub genes screened with five algorithms from cytoHubba and the weighted gene co-expression network analysis. (**A**) Protein–protein interaction network diagram of the LIM domain family genes. (**B**) The Venn diagram revealed that five algorithms screened seven overlapping hub LIM domain family genes. (**C**) Three modules were identified based on the hierarchical clustering dendrogram of 47 LIM domain family genes. (**D**) Heatmap of the correlations between MEs and the trait for the tumor and normal lungs. The MEblue module was significantly positively correlated with clinical traits in the tumor, while the MEturquoise module was significantly positively associated with clinical traits in healthy lungs. (**E**–**J**) Representative immunohistochemistry images of LIM and senescent cell antigen-like domain 1 (LIMS1) across clinical specimens of non-small-cell lung cancer samples in the HPA database.

Our results revealed that the LIM domain family genes were associated with clinical significance. To identify the hub module among the 47 genes, we initially constructed the protein–protein interaction (PPI) network (Figure 6A) and used five algorithms to generate hub genes. We identified seven hub LIM domain family genes that were shared by all the algorithms, including *LHX3*, *Zyxin*, *LMO3*, *LMO1*, *LHX9*, *CSRP1*, and *LIMS1* (Figure 6B). Additionally, we used the weighted gene co-expression network analysis (WGCNA) analysis to construct a module–trait matrix. As indicated in Figure 6C, hierarchical clustering grouped the LIM domain family genes into three modules. The module–trait relationship is illustrated in Figure 6D. We observed that the MEblue module was significantly positively associated with clinical traits in the tumor, suggesting that genes of MEblue were associated with tumor characteristics. In contrast, the MEturquoise module was significantly positively related to clinical traits in healthy lungs. Therefore, the genes in the MEblue module may be related to the phenotypes of NSCLC. Interestingly, LIMS1 belongs to both the hub genes in PPI and the MEbule module. We further examined the expression of LIMS1 protein in NSCLC tissues based on the Human Protein Atlas (HPA) database. As indicated in Figure 6E–J, the LIMS1 protein was strongly expressed in tumor tissues among six patients with NSCLC (three with lung adenocarcinoma and three with lung squamous cell carcinoma).

### 2.6. LIMS1 as a Pro-Tumor Gene in NSCLC

**Figure 7:**
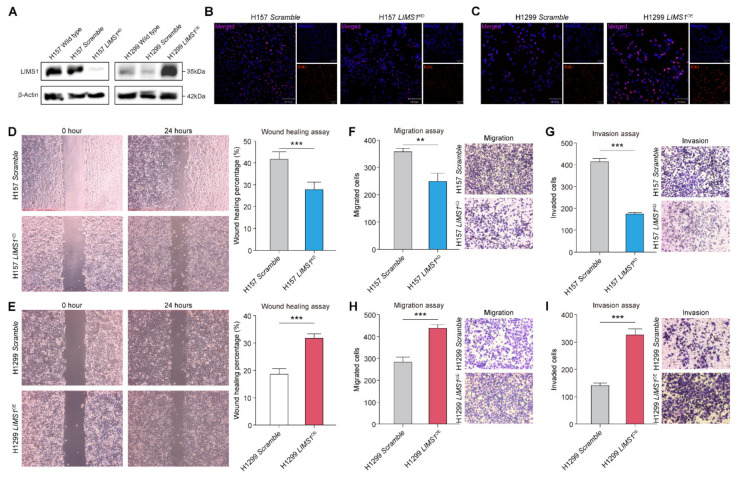
The role of LIM and senescent cell antigen-like domain 1 (LIMS1) in the regulation of non-small-cell lung cancer (NSCLC) progression. (**A**) The effect of LIMS1 knockdown or overexpression was verified with Western blotting. (**B**,**C**) EdU assay measured the effects of LIMS1 on the proliferation potential of NSCLC cells. The images were measured under 100× magnification. (**D**,**E**) The wound-healing assay measured the effects of LIMS1 on the migratory potential of NSCLC cells. The images were measured under 40× magnification. (**F**–**I**) The transwell assay measured the effects of LIMS1 on the migration and invasion potential of NSCLC cells. The images were measured under 100× magnification. ** *p* < 0.01; *** *p* < 0.001.

To further explore the role of LIMS1 in NSCLC, we constructed two in vitro models using the H1299 and H157 cell lines. As indicated in Figure 7A, LIMS1 expression was upregulated or knocked down in H1299 and H157 cell lines, respectively. We used EdU incorporation assays to explore the effects of LIMS1 on cell proliferation. According to the results, compared with that in the control group, the number of EdU^+^ cells in H157 cells with decreased LIMS1 expression was significantly reduced (Figure 7B). In contrast, compared with that in the control group, the number of EdU^+^ cells in H1299 cells with increased LIMS1 expression was significantly increased (Figure 7C), suggesting that LIMS1 promoted the proliferation of NSCLC cells. We also explored the effect of LIMS1 on the migratory capabilities of NSCLC cells via a wound-healing assay and a transwell assay. The wound-healing assay presented that the rate of migrated H157 cells with decreased LIMS1 expression was significantly lower than that in the control group (Figure 7D). In contrast, the migration rate of H1299 cells with increased LIMS1 expression was markedly higher than that in the control group (Figure 7E). The transwell assay also revealed that, compared to the control group, the migrated and invaded tumor cells were decreased in H157 cells with decreased LIMS1 expression (Figure 7F,G). Contrasting results of migration and invasion were observed in H1299 cells with increased LIMS1 expression (Figure 7H,I). Altogether, these results revealed that LIMS1 promoted the proliferation, migration, and invasion abilities of NSCLC cell lines, suggesting that LIMS1 may function as a pro-tumor gene and promote tumor progression.

## 3. Discussion

Increasing evidence has demonstrated that the LIM domain family genes play an indispensable role in tumor initiation, progression, and host immunity [21,22,23,24]. Previous studies have focused on a single gene or single TME cell type; however, the role of the LIM domain family genes in TME infiltration in NSCLC has not been comprehensively recognized. The discovery of the role of the LIM domain family genes in the TME will contribute to the development of more effective treatment strategies. Our study summarized the expression and mutation patterns of the LIM domain family genes based on The Cancer Genome Atlas (TCGA)–NSCLC, and the frequency of global alterations was 33.83%. Using the unsupervised clustering algorithm, we classified patients with NSCLC into two gene clusters. We observed that most LIM domain family genes were more highly expressed in cluster 2, namely, the LIM-high group. Interestingly, patients in the LIM-low group had a better survival rate than those in the LIM-high group. Moreover, the analysis of the TME for both groups revealed a higher proportion of CD8^+^ T cells and follicular helper T cells in the LIM-low group. However, the proportions of MDSC, macrophages, and regulatory T cells were significantly higher in the LIM-high group. Additionally, the LIM-high group revealed a higher stromal score with significance, whereas the LIM-low group exhibited a higher tumor purity. Meanwhile, to further explore the association between the LIM signature and TME in NSCLC, we constructed the LIMscore based on the expression level of 47 LIM domain family genes using PCA. We demonstrated that the LIMscore was negatively associated with CD8^+^ T cells and activated NK cells, albeit positively associated with MDSC, M0 macrophages, and activated mast cells. These results indicated the possibility of an immunosuppressive microenvironment in the LIM-high group. Additionally, the IPS was significantly increased in the LIM-low group, suggesting its higher sensitivity to immunotherapy. Conclusively, there were significant differences in the characteristics of TME between the LIM-low and LIM-high groups, suggesting that the LIM domain family genes can provide reasonable recommendations for personalized immunotherapy for patients with NSCLC.

Several studies have elucidated the potential mechanism of the LIM domain family genes involved in tumorigenesis and progression. In breast cancer, abnormal expression of LMO4 could enhance the transforming growth factor-β (TGF-β) signaling pathway and may promote breast cancer progression by regulating epithelial–mesenchymal transformation (EMT) regulated by TGF-β [25]. Malvi et al. reported the potential mechanism by which LIMK2 promotes the metastatic progression of breast cancer by activating SRSF protein kinase 1 [26]. In prostate cancer, patients with a high expression of PDLIM2 had a poor prognosis, and PDLIM2 was correlated with EMT and immune cell infiltration by acting as an oncogene [20]. LMO2 may also promote prostate cancer progression by inhibiting E-cadherin expression [27]. In colorectal cancer, PDLIM1 could inhibit EMT and the metastatic potential of colorectal cancer cells via stabilizing the E-cadherin/β-catenin complex [28]. The imbalanced expression of LIMK1 and LIMK2 could promote β-catenin nuclear translocation and activate the wnt signaling pathway, thus leading to colorectal cancer progression [29]. Furthermore, FHL3 could promote EMT and chemotherapy resistance via up-regulating Slug and activating TGF-β/Smad-independent pathways, thus leading to metastasis of gastric cancer [30]. The above studies show that the LIM domain family genes are closely related to EMT, suggesting that it may be an important mechanism of the LIM domain family genes involved in tumor development.

Moreover, previous studies have identified the crucial roles and potential mechanisms of LIM domain family genes in NSCLC. Shi et al. found that PDLIM5 contributes to the migration, invasion, and lung metastasis of NSCLC cells. Mechanistically, they demonstrated that PDLIM5 promotes TGF-β signaling and malignance of lung cancer by specifically interacting with SMAD3 and preventing its degradation [31]. Hou et al. discovered that FHL3 promoted the growth, proliferation, and invasion of NSCLC cells [32]. LMO1 can also act as an activated tumor promoter that activates AKT signaling in NSCLC [33]. LIM domain-containing protein 1 (LIMD1) is a tumor suppressor gene occasionally ablated early in lung cancer development [34]. Moreover, LIMD1 is a prognostic indicator for NSCLC, and its loss significantly worsened patient survival [35,36]. LIMS1, a member of the PINCH family, plays important roles in cell–ECM adhesion, migration, proliferation, and survival [37]. Recent studies have reported its vital role in cancer progression. For instance, a high level of LIMS1 promoted tumor progression in breast cancer, and the LIMS1–myoferlin signaling axis may contribute to this process [15]. In skin cancer, LIMS1–neural precursor cells expressed a developmentally downregulated protein 4-insulin-like growth factor-1 receptor signaling axis, which is critical for promoting skin cancer cell proliferation and survival [14]. Guo et al. discovered that LIMS1 is highly expressed in lung adenocarcinoma and promotes proline synthesis, cell proliferation, and tumor growth [38]. In this study, the protein expression of LIMS1 in NSCLC tissues was analyzed in the HPA database, and the results revealed that LIMS1 protein was strongly expressed in NSCLC tissues. Subsequently, we performed some in vitro experiments to explore the function of LIMS1. These results exhibited that LIMS1 expression was positively associated with the proliferation, migration, and invasion of NSCLC cells, indicating that *LIMS1* may function as an oncogenic gene and be a potential target in NSCLC.

Although we performed a comprehensive analysis of the LIM domain family genes and TME infiltration characterization in NSCLC, which lays a foundation for future exploration of NSCLC progression, this study still has some limitations. Since our NSCLC samples were only obtained from retrospective studies based on the TCGA databases, more cases from prospective research are required. Furthermore, the role of LIMS1 was explored in NSCLC cell lines in vitro; however, comprehensive functions of LIMS1 and its relationship to TME remain elusive, which needs to be further explored through in vitro and in vivo experiments and clinical samples. Additional research is crucial to identify the specific molecular mechanisms of the LIM domain family genes regulating NSCLC progression.

## 4. Materials and Methods

### 4.1. NSCLC Dataset and Processing

We downloaded RNA-seq transcriptome data, nucleotide variation data, and the clinical records from the NSCLC (n = 1089) datasets from the TCGA database. Patients without corresponding clinical data were further excluded. We downloaded the RNA-seq data with count value from the Genomic Data Comments (GDC, https://portal.gdc.cancer.gov/, accessed on 12 November 2022) and transformed them as fragments per kilobase of transcript per million mapped read values using R software. We also downloaded the somatic mutation data from the TCGA database and processed it with the maftools package in R software.

### 4.2. Selection of the Differentially Expressed LIM Domain Family Genes

We identified a 47-gene panel as the LIM domain family through retrieval of the relevant literature (Table 1). Further, we compared the expression of these genes in NSCLC tumors with that of normal tissues and identified 43 differentially expressed LIM domain family genes in patients with NSCLC. We used the limma package in R software to process all the data.

### 4.3. Consensus Clustering of LIM Domain Family Genes and Construction of LIM Signature

Based on the expression levels of 47 LIM domain family genes, we used an unsupervised clustering analysis with the optimal k value with the ConsensClusterPlus package in R software to generate gene clusters. Patients with NSCLC were divided into two groups (LIM-high and LIM-low groups). A consensus clustering algorithm was used to determine the optimal number of clusters and their stability. Further, we used PCA to construct the LIMscore based on the expression levels of 47 LIM domain family genes. Both principal component 1 and principal component 2 were selected for calculating the LIM signature scores. Survival analyses were performed using the Survminer and survival packages in R software and visualized as Kaplan−Meier (KM) survival curves.

### 4.4. Functional Enrichment Analysis and Immune Infiltration Analysis

GO and KEGG analyses were performed using the clusterProfiler and org.Hs.eg.db packages in R software based on the DEGs between the LIM-high group and the LIM-low group. The critical value of the false discovery rate (FDR) was <0.05. GSEA was performed using the enrichplot, clusterProfiler, and org.Hs.eg.db packages in R software according to the GSEA algorithm. We used the estimate package in R software to estimate the abundance of the infiltrating immune cells in NSCLC tumors. Additionally, we quantified the relative abundance of each infiltrating cell population in the TME of the NSCLC tumors using single-sample GSEA (ssGSEA) with the gsva package in R software. Using the ssGSEA algorithm, the relative abundance of different infiltrates in each sample was calculated according to the enrichment scores.

### 4.5. Identification of the Hub Genes of the LIM Domain Family Genes

We initially used STRING (version 11.5) to perform PPI network and functional enrichment analyses. Then, the PPI network was exported into Cytoscape software to determine the hub genes of the LIM domain family genes. We used the cytoHubba plug-in to identify the hub genes in the network according to the five different algorithms. Furthermore, we performed WGCNA using the WGCNA and limma packages in R software to determine tumor-related modules and hub genes. Genes were classified into modules based on the topological overlap matrix (TOM)-based dissimilarity measure. The parameters used for WGCNA were as follows: cut height 0.25, soft-thresholding power 9, and minimal module size 7.

### 4.6. Cell Culture and Transfection

Human NSCLC cell lines H1299 and H157 were obtained from the American Type Culture Collection. The Roswell Park Memorial Institute (RPMI)-1640 medium (Gibco, Thermo Fisher Scientific, Waltham, MA, USA) supplemented with 10% fetal bovine serum (Gibco, Thermo Fisher Scientific) and 1% penicillin–streptomycin antibiotic solution was used for cell culturing. The specific small interfering RNAs for LIMS1 and a negative control siRNA were purchased from Ribobio Company (www.ribobio.com, accessed on 5 August 2022). Overexpression of LIMS1 was also conducted with plasmids synthesized in Shanghai GeneChem Company (www.genechem.com.cn, accessed on 4 August 2022) via transfection. The transfection was performed per the manufacturer’s instructions of the Lipofectamine 3000 Transfection Kit (Invitrogen, Thermo Fisher Scientific). Forty-eight hours following transfection, tumor cells were collected for subsequent experiments.

### 4.7. Western Blot

First, H1299 and H157 cells were entirely lysed, and total protein was extracted using RIPA lysis buffer (Beyotime, China). Second, protein loading buffer was added to the protein sample, mixed to a concentration of 1×, and boiled for 8 min. Third, a 30-µg protein sample was added to each well for electrophoresis, which was then transferred to a polyvinylidene fluoride (PVDF) membrane. Last, the membrane was blocked with 5% skim milk for 2 h and subsequently incubated with the primary antibodies (LIMS1/β-Actin, Abcam, Waltham, MA, USA) overnight at 4 °C. Bands were then incubated with the secondary antibody (Abcam, Cambridge, UK) for 2 h at room temperature. The immune complex was detected using chemiluminescence (Amersham Imager 600, General Electric, Boston, MA, USA).

### 4.8. EdU Assay, Wound-Healing Assay, and Transwell Assay

The EdU Imaging Kit (RIBOBIO, China) was used for evaluating cell proliferation following the manufacturer’s instructions. One hundred microliters of 1× Hoechst (RIBOBIO, Guangzhou, China) were used to stain the cell nuclei. Cells with EdU^+^ were visualized using a fluorescence microscope (Olympus, Tokyo, Japan).

The cells were seeded densely in a six-well plate and cultured to confluence. Further, a 200-μL sterile tip was used to scratch a wound line across the monolayer cells. The detached cells were further washed away with phosphate-buffered saline. Cells were cultured in RPMI-1640 and photographed at 0 and 24 h post-wounding. Images were captured using a phase-contrast microscope (OLYMPUS, Japan). Each assay was replicated thrice.

The migration assay was performed with a 24-well transwell chamber without Matrigel (Corning, NY, USA) coated in the upper chamber, and the invasion assay was performed with its upper chamber coated with Matrigel. A total of 2 × 10^5^ (invasion) or 10^5^ (migration) transfected H1299 or H157 cells in 200 µL of serum-free RPMI-1640 was first seeded in the upper chamber. Next, 700 µL of medium containing 10% FBS was added to the lower chamber. Following 24 h incubation, the cells on the upper membrane were carefully removed with a cotton swab. The invaded cells that traversed the membrane were identified with crystal violet staining and photographed. The invaded cells were counted manually and confirmed using ImageJ software (NIH, Bethesda, MD, USA).

### 4.9. Statistical Analysis

Data are presented as mean ± standard error of the mean. Comparisons between groups were performed using Student’s *t*-tests for continuous variables and χ^2^ tests or Fisher’s exact tests for categorical variables. The log-rank test was used to determine the statistical significance of the difference between survival curves. *p* < 0.05 was considered statistically significant. The KM survival curves were plotted using the Survminer package in R software. All statistical analyses were performed using R software and GraphPad Prism 8.

## 5. Conclusions

In this study, a comprehensive assessment of the expression and mutation profiles of 47 LIM domain family genes in NSCLC was performed, and we comprehensively analyzed the role of the LIM domain family genes in TME and immunotherapy. Initially, the patients with NSCLC were stratified into two LIM-related clusters based on 47 LIM domain family gene expression levels: the LIM-high group and the LIM-low group. Further analyses demonstrated that the two groups had different prognoses and TME-infiltrated immune cells, indicating that the LIM domain family genes played crucial roles in the TME of NSCLC. Moreover, we determined that *LIMS1* was the hub LIM domain family gene based on the bioinformatic analysis. Further assays verified that *LIMS1* may act as a pro-tumor gene and promotes tumor progression, thus presenting as a potential biomarker and therapeutic target in NSCLC.

## Figures and Tables

**Table 1:** Members of the LIM domain family genes.

Family	Members
*LHX*	*ISL1*, *ISL2*, *LHX1*, *LHX2*, *LHX3*, *LHX4*, *LHX5*, *LHX6*, *LHX8*, *LHX9*
*CRP*	*CSRP1*, *CSRP2*, *CSRP3*
*FHL*	*ACTG2*, *FHL1*, *FHL2*, *FHL3*
*Paxillin*	*TGFB1I1*
*LIMK*	*LIMK1*, *LIMK2*
*LMO*	*LMO1*, *LMO2*, *LMO3*, *LMO4*
*Enigma*	*PDLIM5*, *PDLIM7*
*MICAL*	*MICAL1*, *MICALL1*
*LASP*	*NRAP*
*ALP*	*ALPP*, *PDLIM1*, *PDLIM2*, *PDLIM4*
*PINCH*	*LIMS1*, *LIMS2*
*Testin*	*PRICKLE3*, *PRICKLE4*, *RILP*
*Zyxin*	*TRIP6*, *WTIP*, *ZYX*, *LPP*, *FBLIM1*, *LIMD1*
Other	*ABLIM1*, *LIMA1*, *LMO7*

## Data Availability

Please contact the corresponding author to discuss availability of the data presented in this study.

## Supplementary Materials

The supporting information can be downloaded at: https://www.mdpi.com/article/10.3390/ijms24054524/s1.
