# Supplementary material for: Comprehensive Analyses and Immunophenotyping of LIM Domain Family Genes in Patients with Non-Small-Cell Lung Cancer
Source: Int J Mol Sci. 2023 Feb 24;24(5):4524. doi: 10.3390/ijms24054524 (PMC10003053; doi:10.3390/ijms24054524)
Supplement: Supplementary file 1 [file ijms-24-04524-s001.zip › ijms-2188324-supplementary/ijms-2188324 Figure S1.pdf]

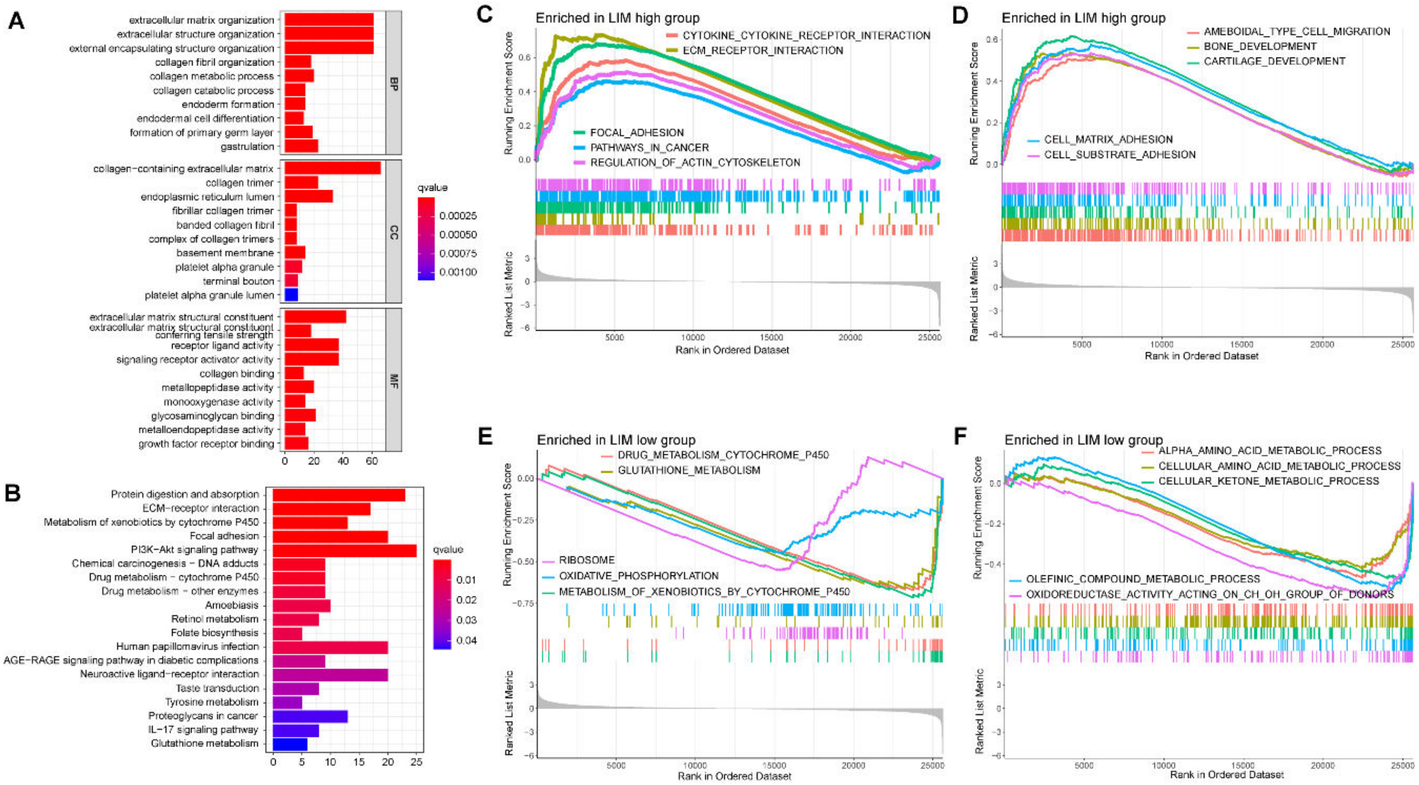

Figure S1. (**A, B**) The GO and KEGG enrichment analyses of differentially expressed genes among the LIM-low and the LIM-high groups. (**C, D**) GSEA revealed the enrichment pathways in the LIM-high group based on KEGG analyses and GO analyses, respectively. (**E, F**) GSEA revealed the enrichment pathways in the LIM-low group based on KEGG analyses and GO analyses, respectively.
